# Comprehensive Evaluation and Future Perspectives of Non-Surgical Contraceptive Methods in Female Cats and Dogs

**DOI:** 10.3390/ani15101501

**Published:** 2025-05-21

**Authors:** Sheila I. Peña-Corona, Melissa Aurea Vaquera-Guerrero, José Cerbón-Gutiérrez, Juan I. Chávez-Corona, Adrián E. Iglesias-Reyes, Alonso Sierra-Reséndiz, Juan José Pérez-Rivero, Socorro Retana-Márquez, Pablo Adrián Vizcaino-Dorado, David Quintanar-Guerrero, Gerardo Leyva-Gómez, Dinorah Vargas-Estrada

**Affiliations:** 1 Departamento de Farmacia, Facultad de Química, Universidad Nacional Autónoma de México, Ciudad de México 04510, Mexico; sheila.irais.pc@gmail.com (S.I.P.-C.); 315331620@quimica.unam.mx (M.A.V.-G.); juan.isaac.chavez@gmail.com (J.I.C.-C.); 2 Departamento de Reproducción, Facultad de Medicina Veterinaria y Zootecnia, Universidad Nacional Autónoma de México, Ciudad de México 04510, Mexico; josecerbon@fmvz.unam.mx; 3 Laboratorio de Investigación y Posgrado en Tecnología Farmacéutica, Universidad Nacional Autónoma de México-FESC, Campus 1, Cuautitlán Izcalli 54714, Mexico; quintana@unam.mx; 4 Departamento de Producción Agricola y Animal, Universidad Autónoma Metropolitana Unidad Xochimilco, Ciudad de México 04960, Mexico; aiglesias@correo.xoc.uam.mx (A.E.I.-R.);; 5 Centro Cultural Ecuestre Domecq, Carretera México Veracruz Kilómetro 30.5, Sta Ines, Texcoco 56240, Mexico; alonsosierraresendiz@gmail.com; 6 Departmento de Biología de la Reproducción, Universidad Autónoma Metropolitana-Iztapalapa, Ciudad de México 09340, Mexico; sretanam@gmail.com; 7 Laboratorio de Medicina Genómica, Departamento de Genómica, Instituto Nacional de Rehabilitación Luis Guillermo Ibarra Ibarra, Ciudad de México 14389, Mexico; adrian_vizcaino@hotmail.com; 8 Departamento de Fisiología y Farmacología, Facultad de Medicina Veterinaria y Zootecnia, Universidad Nacional Autónoma de México, Ciudad de México 04510, Mexico

**Keywords:** contraception, non-surgical, biotechnology, estrus cycle, GnRH, zona pellucida, hormones

## Abstract

The issue of stray cats and dogs is a global concern with considerable implications for animal welfare and public health. This review aims to provide an updated and comprehensive analysis of non-surgical contraceptive methods tested in studies controlled in vivo in feline and canine females. Immunocontraception via vaccination against gonadotropin-releasing hormone (GnRH), the luteinizing hormone receptor, zona pellucida proteins, and sperm, or use of viral-vectored delivery, is yet developing. Hormonal treatment (progestins, androgens, or GnRH) analogs act directly to block the reproductive axis. However, it produced essential side effects. Analogs of kisspeptin, non-steroid anti-inflammatory drugs such as firocoxib, and delivery of cytotoxins to the pituitary have shown non-conclusive results. Additional methods have also been tested, such as intraovarian injection of necrosing compounds or intravaginal and intrauterine devices. At present, neither of these methods offers permanent sterility that can replace surgical sterilization techniques. To our knowledge, none are currently authorized by the Food and Drug Administration (FDA) or the European Medicines Agency (EMA) for contraceptive methods or sterilization of cats or dogs. Therefore, it is necessary to continue the development of a compound that warrants the sterility of cats and dogs.

## Simple Summary

The overpopulation of stray cats and dogs is a public health issue worldwide. Surgery is the most common method of contraception; however, it may be beneficial to have a non-invasive technique for controlling the reproduction of cats and dogs. Therefore, this review offers an updated and comprehensive analysis of non-surgical contraceptive methods tested in controlled in vivo studies involving female felines and canines, focusing on the effects of reproductive variables and the duration of sterility achieved.

## 1. Introduction

There is an urgent need to control the rapidly growing populations of feral cats and dogs worldwide [1]. These species were domesticated 9000 to 10,000 years ago and 40,000 years ago [2]. The original purposes of domestication for these species have shifted to “companion pets” or “domestic animals,” leading to significant overpopulation issues globally and creating a serious public health concern [3]. Approximately 600 million cats and between 700 and 1000 million dogs inhabit our planet, with around 300 million stray dogs and 70 million stray cats [2,3,4,5,6]. In Mexico, the pet dog population was estimated to be around 42 million, while there were about 7 million cats [7].

The number of stray cats and dogs poses a global concern for animal welfare and public health. Some dog breeds have lost their ability to survive independently of humans, increasing the risk of disease transmission and reports of aggression. Feral cats can have an ecological impact that harms native wildlife, threatening 8% of endangered birds, mammals, and reptiles [6]. Therefore, controlling the population of cats and dogs relates to improving various aspects of society.

As a result, the World Organization for Animal Health (WOAH) has stated that controlling the population of these species is necessary [8]. It is estimated that sterilizing 76–88% of female dogs would be required [9] to halt the growth of the canine and feline populations. Canine and feline population control methods are categorized into surgical and non-surgical types. Surgical methods, such as TNR (Trap–Neuter–Return), are the most widely practiced and accepted globally. However, they have several disadvantages that significantly complicate their implementation.

Surgical contraception is a costly method that may lead to complications such as hemorrhage, bloody vulvar discharge, the onset of pseudocyesis, infection at the incision site, and peritonitis [10]. Additionally, pediatric sterilization is a controversial topic, and its effects are still being studied. Authors like Oliveira et al. (2023) and McKenzie (2010) note that this procedure can cause behavioral changes such as increased fear and shyness, and it may also predispose individuals to conditions like cranial cruciate ligament rupture or a higher incidence of cystitis [11,12,13]. Furthermore, the risks may be heightened if the female is in heat during surgery, as well as due to her age, health status, and sensitivity to drugs or anesthetics [14,15,16].

Therefore, surgical contraception is not the better method for controlling stray populations of felines and canines. Non-surgical options include immunocontraception targeting gonadotropin-releasing hormone (GnRH) or the zona pellucida (ZP), hormone analog implants like deslorelin acetate, suppression of genes associated with GnRH release, and intrauterine devices. These methods avoid the adverse effects of surgery, do not require sedation, and eliminate the need for post-operative care [8,15,17].

Currently, there are no effective non-surgical contraception methods without side effects that provide long-lasting results. Consequently, this field is continuously evolving. This review offers a thorough and updated analysis of non-surgical contraceptive methods evaluated in controlled in vivo studies on female felines and canines. It highlights the impact of reproductive variables, the duration of sterility achieved in females, and the evaluated protocols and doses as the side effects while addressing current challenges and future research opportunities.

## 2. Materials and Methods

**Figure 1:**
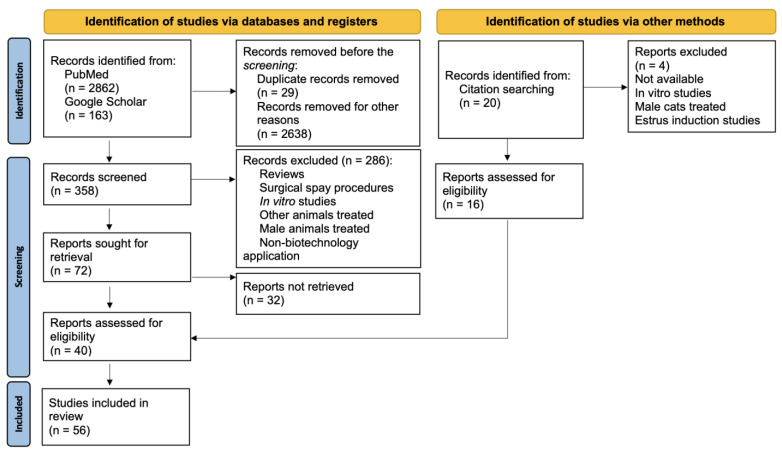
PRISMA flow diagram.

As a tool for searching for contraceptive methods used in female canines and felines, we used the PRISMA flow diagram (Figure 1). The search was performed in PubMed and Google Scholar from February 2024 until April 2024. To obtain broad information about contraceptive methods, we consider all the dates on which the articles were published. The topic keywords were established using the PECO principle from the PRISMA statement [18]. The study population [P] consisted of female cats or dogs subjected to in vivo exposure [E] to contraceptive drugs. The comparator [C] group consisted of animals that received a vehicle-only treatment (the control group). The primary outcome [O] was the suppression of fertility, which was evaluated at specific doses and time points (days post-administration) to assess the efficacy of contraceptive drugs.

The terms searched were generally the following: contraception AND non-surgical AND female cats AND/OR bitches; biotechnology; vaccines, hormone, gonadotropin-releasing hormone (GnRH), corpus luteum, kisspeptin, estrus cycle, exfoliative cytology, female. Furthermore, filters of species and sex were used in PubMed, and the combinations were used to obtain more specific results. Additionally, references from other reviews and original articles were checked manually. The articles were selected based on the following criteria:

- English articles about non-surgical spaying based on biotechnology;
- Studies in vivo involving female canines or felines.

Reviews and non-English articles, which include surgical spay and in vitro or situ trials, utilized models other than female felines or canines, which were excluded.

Two reviewers conducted the screening process. One reviewer read the titles and abstracts to pre-select articles, while another evaluated the retrieved articles individually to exclude duplicates or those not meeting the inclusion criteria. Finally, the screening was completed by reading the full article texts.

## 3. Results

Our research initially identified 2862 studies from PubMed and 163 from Google Scholar, with approximately 56 articles selected for inclusion based on their relevance to the research topic (Figure 1). As the initial search progressed, we discovered methods tested in bitches to induce the contraceptive state through non-surgical procedures, vaccines, hormone analogs, modulators of the corpus luteum (CL), kisspeptin (KP), ultrasonic exposure, and zinc gluconate. In cats, vaccines and hormone analogs have also been tested. In this section, we describe our search results (Table 1) and explain and discuss the findings of the contraceptive methods in the subsections.

### 3.1. Immunocontraception

**Figure 2:**
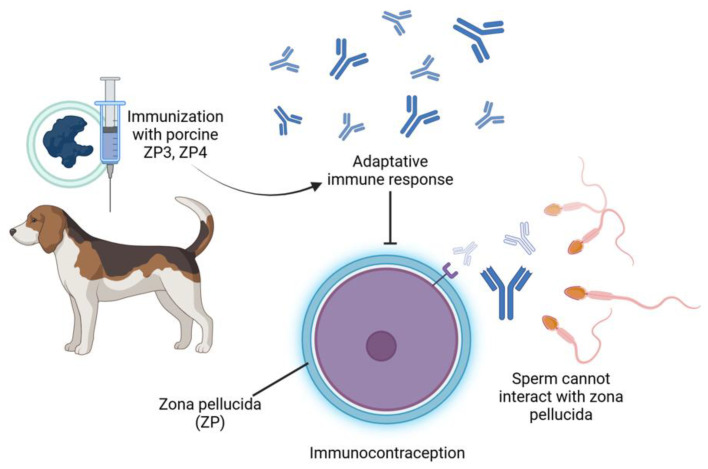
Mechanism of immunocontraception in female dogs using porcine ZP3 and ZP4. Immunization with porcine zona pellucida proteins (ZP3 and ZP4) triggers an adaptive immune response in the female dog.

Vaccines aimed at reproductive processes represent a method of nonlethal control over abundant free-roaming species [18,23]. Immunocontraception stimulates the production of antibodies that neutralize and block hormone receptors, disrupting the estrus cycle by inhibiting critical proteins or hormones required for reproduction [20,24], such as GnRH (which prevents ovulation by blocking the release of gonadotropins) [23] and zona pellucida (ZP) (which inhibits fertilization and folliculogenesis) [21]. A booster is necessary to prolong the infertile period, ensuring a sustained effect for approximately one year [36,37]. These methods are safe and generate high titers of antibodies for at least one year in female cats and dogs (Figure 2).

The most common antigen in vaccines is GnRH, conjugated to KLH (keyhole limpet hemocyanin) as a carrier in the commercial vaccine GonaCon^®^ (National Wildlife Research Center NWRC, Fort Collins, CO, USA) or as a recombinant protein (e.g., in eight tandem repeats or coupled to a fraction of ZP) [25]. This is safe and to produce a high antibody titer for at least one year in female cats and dogs [23,25,34]. In bitches, the use of sperm as an antigen has led to the development of specific anti-sperm antibodies. However, three immunizations are necessary to achieve a higher titer of antibodies [35]. Another frequently used antigen is porcine zona pellucida (pZP), which induces a high titer of antibody production in bitches and cats. Eade et al. (2009) [19] evaluated the contraception in cats resulting from immunization with ZP polypeptide (55 kDa) and feline ZP A, B, and C subunits expressed by plasmid vectors. The authors concluded that the administration of feline ZPA and ZPB+C subunits are potential candidate antigens for immunocontraceptive vaccines in domestic cats [19]. Immunization of female dogs with bovine luteinizing hormone receptor (LH-R) immunomodulates ovarian function, resulting in a reversible state of infertility [2]. Given the results described, using vaccines provides a reliable method to promote reversible contraception; however, no vaccines guarantee infertility for an extended period (Table 2).

As with cats and dogs, overcoming tolerance to self-antigens and inducing durable immunity without repeated booster vaccines is essential for improving the development of immunocontraceptive vaccines [23]. It is also crucial to consider the risk of autoimmune diseases arising from immunization [25] and to manage the duration of contraception, which can vary. In some studies, the vaccines were ineffective during existing pregnancies in cats [20,21,24], while others reported effective durations ranging from days and months to five years [21,23] (Table 2).

Despite this, vaccines that can maintain their long-term antigenic effect are still being developed, as well as devices that allow good antigenic absorption for a long time and with a single administered dose of the antigen without harming the animals [73,74]. Studies are also being carried out in other animals such as mice, where, although the total fertility of the animals has not been eliminated, the number of up to five specimens per litter has been reduced in mice from the experimental groups to which the antigen was inoculated, either in the quadriceps femoris muscle or intradermally [75,76] (Table 2).

The SPRASA protein has also suggested an advance in this method since it only develops in sperm and oocytes of fertile men and women, having an essential function in fertility, and creating a specific antibody would decrease the fertility of individuals [77].

**Figure 3:**
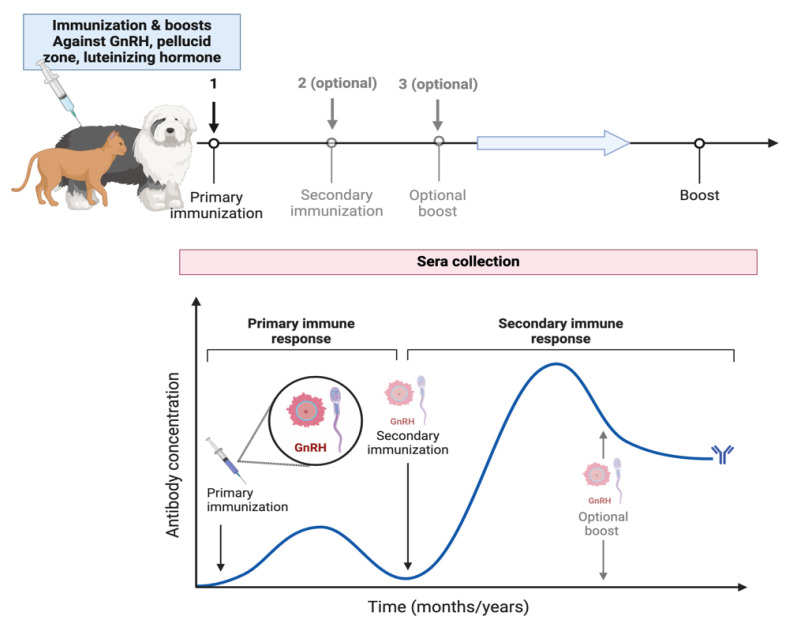
Canine and feline contraception through immunization and booster vaccines targeting GnRH, the pellucid zone, and luteinizing hormone. It illustrates the timeline and process of primary vaccination followed by optional secondary immunizations and optional boosts, leading to a final boost. The graph below represents the antibody concentration over time, highlighting the primary immune response initiated by the first immunization and a secondary immune response after subsequent immunizations. The antibody concentration increases with each vaccine, peaking during the secondary immune response and declining until the next boost.

In some other studies, contraceptive vaccines for animals have been explored, which can be divided into regulators of gamete production and regulators of gamete function. Within the first group are GnRH, FSH, and LH [78]. Cats were inoculated with vaccines containing phages made of chemical conjugates of multiple copies of GnRH causing reduced release of gonadotropin hormones and gonadal atrophy, observing a decrease in epididymal vacuolization and an increase in abnormal sperm [79]. However, the authors recommend its application at an early age (prepubertal), and it may apply to other species (Figure 3) (Table 2).

The practical application of immunizations as a strategy for birth control in large populations remains a subject of debate, mainly due to inherent limitations regarding their efficacy and safety. Firstly, although contraceptive vaccines represent a non-surgical alternative, their implementation should not be considered a primary option. This is mainly because uncertainties persist concerning the potential health risks associated with these immunizations. Such risks cannot be regarded as secondary to the goal of inducing infertility—especially when compared to surgical methods, which offer permanent cessation of reproductive capacity, along with possible additional benefits such as the prevention of diseases linked to hormone-driven sexual stimulation. Secondly, the outcomes associated with the use of contraceptive vaccines show considerable variability. To date, absolute infertility has not been guaranteed, even under protocols involving periodic revaccination. This lack of predictability significantly limits their utility in large-scale population control programs. For these reasons, the implementation of contraceptive immunizations must be accompanied by comprehensive longitudinal studies, including extended follow-up periods of individuals receiving these immunogens, to assess both long-term efficacy and safety.

### 3.2. Hormone Analogs 

Steroid hormones like progestins, estrogens, and androgens have been utilized as reproductive inhibitors in cats and bitches, yielding variable results, and they represent the most evaluated non-surgical method (Table 3).

**Figure 4:**
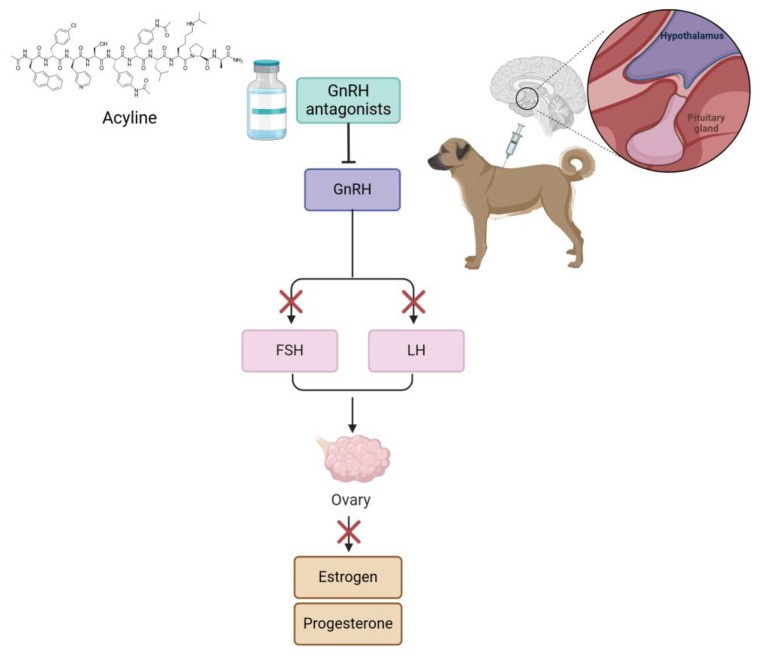
The mechanism of action of GnRH antagonists in a dog model. The process begins with administering acyline, a GnRH antagonist, which blocks the GnRH receptor in the pituitary gland. This inhibits the secretion of FSH and LH, which are normally produced in response to GnRH stimulation. As a result, ovarian production of estrogen and progesterone is suppressed, thereby impacting the reproductive cycle.

Numerous studies have explored drugs that function as agonists, analogs, or antagonists to GnRH receptors to induce contraception in female animals (Figure 4). These drugs disrupt the signaling pathways responsible for hormone synthesis and release, delaying estrus or puberty and extending anestrus periods. Deslorelin acetate is a decapeptide that differs from GnRH by only two amino acids. It acts as an analog of GnRH, exhibiting greater potency at GnRH receptors. In cats, deslorelin acetate has been tested [26,30]. In all articles, reversible alterations in ovulation were observed. In cats, melatonin plays a critical role in regulating the estrus cycle. GnRH antagonists, such as acyline, competitively block the GnRH receptors in the pituitary, inhibiting the activity of the gonadal axis; they also exert an immediate effect compared to agonists [59]. The application of GnRH analog or agonist implants serves not only as a contraceptive method in adult animals but also can delay the onset of puberty in cats and dogs. In a study by Risso et al. (2012) [80], 30 prepubertal crossbreed female domestic cats were divided into two groups of 15. The animals were kept under a photoperiod of 14 h of light and 10 h of darkness. One group received a subcutaneous implant of 4.7 mg deslorelin acetate, while the control group remained untreated. The study aimed to compare the ages at puberty onset between the two groups. The results indicated that the deslorelin-treated group reached puberty between 180 and 428 days, whereas the control group reached puberty earlier, between 134 and 286 days. Deslorelin-treated ovaries appeared small, while control gonads were normal [80] (Table 3).

As GnRH, synthetic steroids can also delay puberty. Medroxyprogesterone acetate (MPA) has been tested for this purpose in female cats. In a study by Lopez et al. (2016) [29], postnatal female kittens (n = 10) were reared freely in indoor catteries with 14 h of light per day and were weaned at 40 days. The kittens were randomly assigned within the first 24 h of birth to receive either 10 mg of MPA per animal (n = 6) or a placebo of 0.2 mL of corn oil (n = 4), administered subcutaneously. During puberty, a mating trial was conducted. Twenty-four days after the end of estrus or ovulation, the kittens were ovariohysterectomized. Ovulation occurred in four of the six MPA-treated kittens and three of the four placebo-treated kittens after estrus, leading to pregnancy. All pubertal kittens displayed normal sexual behavior and accepted repeated matings when exposed to males during estrus [29]. In mature bitches, administration of 4–12.5 mg/kg or 2–12.5 mg/kg of chlormadinone acetate (synthetic progesterone) was conducted for at least 1 year [56]. Additionally, melatonin administration has been used to suppress estrous cyclicity in queens temporarily. Melatonin inhibited ovulation and extended the duration of the interestrus interval (2 to 4 months) [28] (Table 3).

Around nineteen articles focused on the use of these drugs in female dogs. GnRH agonists such as nafarelin or deslorelin mimic their action when used in sustained applications, as they stimulate the production and release of gonadotropins s [26,41,81]. The application of these drugs in bitches during the anestrus phase induces a fertile estrous cycle (flare-up effect). Subsequently, it inhibits the gonadal axis, followed by ovarian quiescence due to the downregulation of GnRH receptors [82]. One of the most studied drugs in bitches is the Suprelorin^®^ implant containing deslorelin acetate, which, with periodic administration, reversibly delays estrus in anestrus females and puberty in prepubertal females for an extended period, depending on the implant dose; for instance, the effect period of the 4.7 mg dose is shorter than that of the 9.4 mg implant. When the implant is removed, the estrous cycle returns to normal [45].

Combinations of Suprelorin^®^ with other drugs have also been tested and have shown favorable responses. For instance, Suprelorin^®^ combined with megestrol acetate (MA) results in a more extended interestrus period [44]; however, the combination with acyline (ACY) does not yield a significant difference compared to Suprelorin^®^ treatment alone [59] (Table 3). Lacoste et al. conducted a study involving nine female beagle dogs that received daily subcutaneous injections of 100 μg [D-Trp6, des-Gly-NH210] GnRH ethylamide dissolved in 0.9% NaCl–1% gelatin for 23 months. This treatment inhibited sexual maturation, but normal pituitary–gonadal functions resumed after cessation, returning to normal within two months post-treatment. Secondary follicles developed following a 14-month recovery period [49] (Table 3).

The continuous administration of synthetic steroids, such as chlormadinone acetate (CMA), in bitches can prevent estrus and delay it for at least one year [56]. As compiled in the present paper, hormone analogs have demonstrated effective results in most cases. Despite their contraceptive efficacy, these hormones can induce side effects that may be life-threatening, including pyometra, mammary fibroadenomatosis, neoplasms, and insulin resistance [28]. In a study conducted by [83] Tamada et al. (2003), estrus was prevented with weekly oral administration of 2 mg chlormadinone acetate for 2.0 to 9.8 years in bitches and queens. Although effective, abnormalities such as mammary or uterine disorders, or both, were observed in seven out of 14 bitches and nine out of 24 queens during this long-term treatment [83] (Table 3).

Progestins are widely investigated due to their progesterone-like structure, which suppresses gonadotropin secretion. When applied, they can cause side effects such as an increased risk of diabetes, tumors, and breast hyperplasia, which is why their use is not yet permitted [77] (Table 3).

In vitro cell cultures have designed peptides with a high capacity and specificity to bind to LH, which administers auristatin to Leydig cells, and another peptide targeting FSH that administers menadione to Sertoli cells. These peptides induce apoptosis of germ cells and can achieve non-surgical sterilization in individuals with a single administration. However, the variation in responses from these experiments remains significant, indicating that further investigation is warranted [84] (Table 3).

Another possibility that has been considered is GnRH hormone agonists produced through amino acid substitutions of the hormone, which grant greater potency and a longer half-life. These agonists negatively regulate the hypophysial GnRH receptors by inhibiting their production, thereby postponing puberty in individuals. For this purpose, postnatal deslorelin in dogs delays puberty in both females and males [85] (Table 3).

Reproductive endocrinology is a well-established and widely utilized field within animal health, holding significant potential to completely inhibit reproductive capacity in this species. However, such interventions must be approached with caution due to the inherent risks associated with manipulating the neuroendocrine axis. The administration of substances that interfere with the synthesis of sex hormones impacts the production of male and female gametes, as well as a range of physiological processes essential for the organism’s overall metabolic function.

Among the most notable consequences is an increased predisposition to insulin resistance and a reduced capacity for synthesizing key neurotransmitters, ultimately disrupting the neurochemical and functional balance of the nervous system. Although hormonal strategies for reproductive control in animals are promising, their implementation must be carefully considered in light of their potential impact on overall health. The sustained alteration of sex hormone levels may lead to systemic pathologies that compromise the organism’s development and well-being, underscoring the complex interconnection between reproductive function and broader physiological systems.

### 3.3. Use of Modulators of Corpus Luteum (CL) as Contraceptive Methods in Bitches

Disrupting the estrus cycle in bitches through CL regulation has been investigated for non-surgical contraception. Trilostane is a drug that disrupts CL function by competitively inhibiting the 3β-hydroxysteroid dehydrogenase/isomerase system (3β-HSD), which is expressed in luteal cells to synthesize progesterone. When administered during the luteal phase, trilostane resulted in a decrease in progesterone levels without affecting ACTH or prolactin levels [62] (Table 4).

Bromocriptine and cabergoline administration affect the maintenance of the CL. Bromocriptine causes an abrupt decrease in progesterone levels [61], and cabergoline inhibits the production of prolactin, a hormone that helps maintain the function of progesterone released by the CL [65]. The CL plays a vital role in the estrus cycle by inducing progesterone production, and previous studies have shown that prostaglandins serve as essential regulators of the CL [63,64,66]. Daily administration of firocoxib, a selective cyclooxygenase-2 inhibitor, to females after ovulation (around the diestrus stage) has been demonstrated to cause, on average, a 10-day suppression in the expression of messenger RNA (mRNA) of prostaglandin E2 synthase, leading to decreased levels of prostaglandins E2 and 2α. The steroidogenic acute regulatory (STAR) protein and prolactin receptor (PR) are other proteins that are not expressed and regulate the CL [64]. Research indicates that if non-surgical sterilization is desired, further investigation can be conducted on the SLC2A1/GLUT1 receptors and the insulin receptor (INR), as these contribute energy to the CL and are related to vasculogenesis.

In bitches, luteotropic modulators offer a potentially safer therapeutic alternative for interrupting the estrous cycle. This method may help avoid adverse effects associated with the direct manipulation of primary neuroendocrine axes, as its action is limited to maintaining the corpus luteum. Although any form of reproductive cycle interruption or manipulation without a clinical indication may pose risks to the organism’s homeostasis, in this context, a strategy that halts the progression of the estrous cycle without causing significant systemic alterations or triggering a pathological condition is preferred.

### 3.4. Kisspeptin (KP)

**Figure 5:**
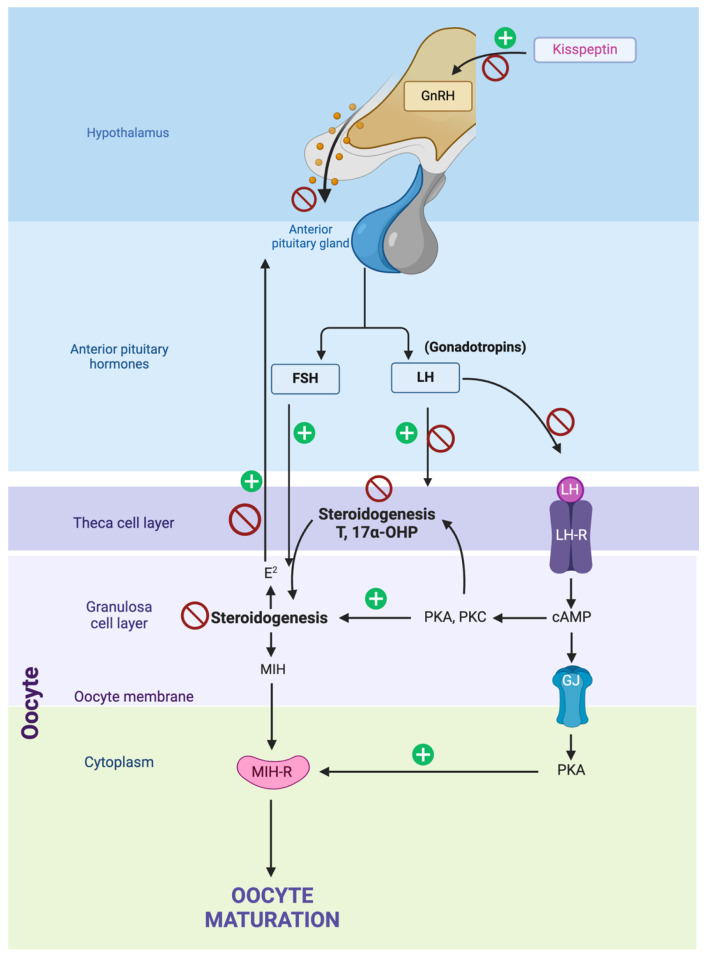
The hypothalamic–pituitary–gonadal axis and the inhibition points tested for canine reproduction inhibition are represented by red stop symbols. Inhibition of kisspeptin, which positively regulates GnRH (gonadotropin-releasing hormone) secretion in the hypothalamus, blocks the initial signal necessary for gonadotropic hormone production. Direct inhibition of GnRH in the hypothalamus prevents the release of FSH (follicle-stimulating hormone) and LH (luteinizing hormone) from the anterior pituitary gland. Inhibiting LH at the pituitary gland level prevents the stimulation of theca and granulosa cells in the ovaries, reducing the production of essential steroids like testosterone (T) and 17α-OHP (17α-hydroxyprogesterone) required for oocyte maturation. Inhibiting FSH in the pituitary gland disrupts the stimulation of granulosa cell activity, affecting estradiol (E2) production and other factors needed for oocyte maturation. Finally, inhibition of steroidogenesis in the theca cell layer reduces the production of testosterone and 17α-OHP, crucial precursors for estradiol synthesis in granulosa cells, thus impacting oocyte maturation.

In 2003, it was discovered that KP signaling serves as a gateway to reproductive function in both males and females. This discovery marked a milestone in unraveling the endocrinological regulation of reproduction [86,87] (Figure 5). The *KISS1* gene encodes KP and its receptor (GPR54 or KiSS1R). When they bind, they trigger the release of GnRH, which travels through the hypothalamic–pituitary system to the anterior pituitary and stimulates the secretion of LH and FSH. This makes it vital in regulating the hypothalamic–pituitary–gonadal (HPG) axis. They also promote the release of LH and other hormones related to the estrus cycle and ovulation, such as E2 and FHS [88]. Therefore, various studies have assessed their role as crucial molecules in controlling the feline and canine estrous cycle [69] (Table 5).

The *KISS1* gene encodes a 145-amino-acid peptide that can be cleaved into four peptides, each containing a common C-terminal RF-amide decapeptide (KP-54, KP-14, KP-13, and KP-10) [89,90]. According to the data compiled in this review, KP-10 is the most tested peptide in female cats and dogs as a contraceptive, yielding variable results (Table 5).

The administration of KP-10 to anestrous bitches rapidly induced significant serum concentrations (Tmax ≤ 5 min, CPmax ≤ 30 min) of hormones associated with the estrous cycle (LH, FSH, E2), with effects lasting up to fourteen days [69]. Terse et al. (2021) observed in dogs that administration of 1000 μg/kg of KP-10 intravenously for 14 days did not induce changes in clinical signs, body weight, hematological values, urine, body temperature, food or water consumption, or sinus rhythms, considering it a safe compound. Therefore, bitches remained in the anestrus phase throughout the entire dosing period [70] (Table 5).

The specificity between ligand and receptor has allowed for investigating antagonist substances with varying responses across the species under study. The continuous intravenous infusion of KP antagonists (p271, p354, and p356) did not result in significant alterations in basal plasma LH concentrations in bitches, into which canine KP-10 was administered two hours after the infusion began [68] (Table 5).

The KP-10 molecule has shown the most significant inhibitory effect during the estrous cycle of female cats and dogs compared to hormone treatments. In other species, its administration has also demonstrated alterations in reproductive processes. The KP-10 analog peptide 234 (p234) exhibited a strong inhibitory effect. Intracerebroventricular administration in rats resulted in delayed vaginal opening (an indicator of puberty) and did not permit serum LH levels to rise when co-administered with KP-10. In intact rats and mice, it required combination with penetratin (p271) to prevent the increase in circulating LH in castrated rodents and sheep [70,91,92] (Table 5).

The administration of agonists and antagonists presents intriguing therapeutic possibilities for addressing endocrinological needs in mammals, such as controlling canine and feline overpopulation, by developing minimally invasive pharmaceutical forms to suppress reproductive capacity temporarily or permanently. However, it remains essential to standardize the dosage and the timing of administration during the estral cycle (Table 5).

It is essential to mention that using vaccines with the *KISS1* gene in sheep, conjugated with the *HBsAG-S* gene, has already reduced sexual behavior and spermatogenesis. Likewise, utilizing the *kisspeptin-54* gene could produce humoral antibodies that neutralize endogenous kisspeptin, thereby reducing testicular growth, scrotal circumference, and spermatogenesis, making it a potential option for non-surgical sterilizations [93] (Table 5).

Alteration of the hypothalamic–pituitary–gonadal axis affects not only reproductive physiology but also a wide range of systemic functions regulated by sex steroid hormones, whose disruption can compromise overall animal health. In this context, modulation of the kisspeptin/GPR54 system represents a promising pathway for reproductive suppression; however, its influence extends beyond reproduction and may lead to broader metabolic and endocrine disturbances. Despite its potential as a contraceptive tool, there remain significant gaps in knowledge regarding the appropriate dosage, route of administration, and safety profile of kisspeptin-based interventions in domestic animals. Therefore, until standardized protocols are established to ensure both efficacy and safety, it is advisable to prioritize contraceptive strategies that do not involve direct suppression of key neuroendocrine axes.

### 3.5. Other Contraception Methods

The effect of therapeutic ultrasound exposure on the ovaries was evaluated, revealing changes indicative of potential fertility loss. Ultrasound treatment (1 MHz frequency, 1.5 W/cm2) applied to dog ovaries for 5 or 10 min heightened the circulating inflammatory response and oxidative stress, leading to a reduction in the number of preserved follicles and oocytes, as well as tissue alterations and changes in ovarian dimensions. However, the results remain inconclusive, necessitating further exploration of the effects of direct exposure of the ovaries to therapeutic ultrasound utilizing different frequencies and power levels [72] (Table 6).

There are commercial spermicides and intrauterine devices for dogs; however, they are not recommended. Reports indicate that these barrier systems are impractical due to the challenges associated with the transcervical cannulation necessary for their application. Other contraceptive methods being tested include those that act with Müllerian inhibitory substances (MISs), also known as anti-Müllerian hormones (AMHs). In one study, AAV9-MIS treatments were placed in female mice that later mated with breeding males and obtained litters very similar to those of the group that did not receive MISs. However, after 6 weeks, the females treated with high AAV9-MISs became infertile, which suggests that this contraceptive method can be beneficial in dogs and cats [94].

Another method used is the administration of zinc gluconate in the ovaries, an invasive treatment that yields favorable results. A study conducted by [71] Mogheiseh et al. (2017) involved healthy, fertile female dogs aged 1–2 years in anestrus or diestrus (n = 5) and evaluated the effects of zinc gluconate injection on the ovaries. A sterile, neutralizing zinc gluconate solution (13.1 mg/mL) was injected into the ovaries, with the volume determined by ultrasound measurements of ovarian diameters. Under general anesthesia, a laparotomy was performed for the injection. One month post-injection, the diameter of the ovaries was significantly reduced, and leukocytes in vaginal cytology decreased from the second to the third day. Histopathological examination revealed hyperemia, fibrosis, and hemosiderin pigment in the ovaries, indicating that not all ovarian structures were destroyed after one month [95]. The application of zinc gluconate administration in females is limited. It requires surgical procedures and the development of protocols to evaluate the side effects of intraovarian administration. It still does not seem to be affordable to the population at this moment because it requires direct contact or administration in ovaries through a laparotomy, which involves the same risks as an ovariohysterectomy. Therefore, it is necessary to try more accessible and non-invasive ways to carry out these treatments.

In recent years, targeted gene silencing has been utilized to induce permanent sterility to control feral populations [96]. Current research on contraceptive vaccines focuses on using adeno-associated virus (AAV). This non-enveloped virus can be engineered to deliver DNA to target cells [97]. Vansandt et al. (2023) [1] utilized AAV9 to provide a female cat with an anti-Müllerian hormone (AMH) transgene (designated fcMISv2). Researchers have demonstrated that the vectored contraceptive prevents breeding-induced ovulation, leading to complete infertility [1] (Table 6).

The implementation of innovative tools in contraceptive methods, encompassing both hormonal and non-hormonal options, has the potential to create new strategies that expand the current perspective on contraception. In this context, the integration of technologies like ultrasound-based contraception signifies a promising area of research, offering a non-invasive alternative that avoids the risk of causing systemic hormonal dysregulation. Furthermore, the development of new hormonal options that do not directly interfere with the endocrine system may encourage the generation of applied knowledge with significant clinical implications. However, these emerging strategies still require a solid experimental foundation and more substantial scientific evidence to uphold their efficacy and safety, allowing them to compete with established methods in the field of contraception.

## 4. Final Considerations

The challenge of suppressing fertility in cats and dogs has existed for over 50 years. In recent years, the interference of the estral cycle through the block of its regulating molecules has been tested (Figure 5), since the regulation of the production of KP [69], until AAV9 was used to prevent breeding-induced ovulation [1]. The primary factor that disrupts the regulation of the estral cycle in female cats and dogs is the involvement of numerous molecules in its control, making it challenging to manage the positive or negative feedback mechanisms that inhibit a particular molecule or signaling pathway.

Technological advances have allowed us to develop more contraceptive-specific methods with fewer side effects. According to the World Small Animal Veterinary Association, an ideal contraceptive product would have the following characteristics: (i) rapidly induce permanent sterilization, (ii) eliminate breeding behavior and fertility, (iii) require only a single dose, (iv) be effective for male and female dogs and cats of all ages, and (v) be safe and easy to administer. The global need for a contraceptive method for feral populations has led to the establishment of prizes and grants, such as The Michelson Prize and Grants. This program aims to prevent the euthanasia of healthy, adoptable companion animals in shelters and reduce the populations of feral and free-roaming cats and dogs. It offers a USD 25 million prize for developing a non-surgical sterilant that can effectively sterilize dogs and cats in a single treatment.

Currently, no product meets all of these criteria, and government institutions authorize none for contraceptive methods or the sterilization of cats or dogs. The tested non-surgical contraceptive methods in female cats and dogs have shown variable results, including immunocontraception, hormonal treatments, kisspeptin analogs, non-steroidal anti-inflammatory drugs, and cytotoxins. However, some promising techniques exist, such as gene therapy, small interfering RNA to inhibit reproductive targets, and the delivery of cytotoxins to pituitary gonadotrophs or GnRH-producing neurons in the hypothalamus. Continued research and innovation in non-surgical population control procedures are necessary, as they are safer for females, less costly, and quicker, allowing for administration to more females in a shorter time frame.

Surgical sterilizations are usually performed at five to seven months of age in bitches, as they have reached sexual or hormonal maturity; it is not suggested that they be performed earlier because bitches may present infantilism; however, it is not suggested that they be performed after the first estrus, as the possibility of mammary gland tumor incidences increases up to seven times [98].

Among the different surgical sterilization techniques, ovariohysterectomy (OVH) midline is the technique most used in the daily practice of veterinary medicine; in this technique, an incision is made in the skin of the ventral midline 4–6 cm below the navel and extending caudally. All these procedures are expected to use a 2–0 polyglycolic acid suture, which will depend on the tissue’s friability, the patient’s size, and the stage of the estrous cycle of the female. With this technique, the hormonal action of the ovaries and uterus is eliminated totally and permanently [98]. In the ovariectomy, only the ovaries are surgically removed, keeping the body of the uterus. It can be performed through two types of approaches. The first and most used is through the midline or lateral ovariectomy, which is a variant of the previous technique [15,99,100,101]. The third most common technique is tubal ligation. One of the advantages of this technique is that it is not very invasive and quick. Still, it does not prevent secondary hormonal reproductive problems, and the females continue their normal sexual cycle [15].

It is essential to mention that wound-healing complications are among the side effects of these types of surgeries. This issue is related to the duration of the surgery, with a greater amount of post-surgical inflammation and infections occurring in procedures lasting more than 90 min. Another common side effect is hemorrhage, which is the most frequent complication in ovariohysterectomies for females over 25 kg, potentially leading to hypovolemic shock and the animal’s death. Stump pyometra is another issue that can arise from these surgical procedures if the ovary is not entirely removed, which may cause the bitch to experience hormonal peaks of endogenous progesterone. Ureteral ligation is also a possible complication from accidentally ligating a ureter during the ligation of the uterine corpus or ovarian arteriovenous complex. Additionally, vaginal bleeding cannot be disregarded as a surgical side effect, and if it becomes severe, celiotomy may be indicated [99,100].

A primary factor in the varied results of non-surgical contraceptives included in this review is the diversity of breeds and sizes of cats and dogs, along with the influence of keeping bitches together, as they may have synchronized estrous cycles [95,102]. Long-haired breeds of cats seem more sensitive to daylight than short-haired breeds [102]. There are distinctions between long- and short-haired cat breeds; for example, Persian cats do not have regular estrous cycles, even during extended daylight. In contrast, short-haired Siamese and related breeds have estrous cycles year-round, regardless of daylight length [102].

Regarding dogs, factors intrinsic to species, such as seasonality, have influenced the results of studies focused on reproduction [103]. The study populations include free-roaming, mixed-breed, purebred, and laboratory dogs. Thus, the diversity of animals examined in studies assessing contraceptive methods may be a crucial element contributing to bias in the results.

## 5. Conclusions

There is an urgent need for effective non-surgical contraceptive methods to manage the increasing populations of stray cats and dogs, which raise significant concerns for animal welfare and public health. While surgical sterilization is the most common form of contraception, researchers are actively exploring non-invasive alternatives, including immunocontraception, hormonal treatments, and targeted gene silencing. These non-surgical methods circumvent surgery-related complications, such as the need for anesthesia and postoperative care. However, none of the current techniques offer permanent sterility or a universally accepted solution, and most have demonstrated limited efficacy with varying outcomes. It is crucial to develop treatments with long-lasting effects, minimal side effects, and the potential for widespread application. Therefore, further research is necessary to enhance these techniques and make them suitable for controlling the overpopulation of stray animals.

## Figures and Tables

**Table 1:** Articles that accomplish the inclusion criteria in the search.

Contraceptive Method (Number of Articles)	References
**Female Cats**	
Vaccines (8)	Eade et al., 2009 [19]; Fischer et al., 2018 [20]; Gorman et al., 2002 [21]; Ivanovo et al., 1995 [22]; Levy et al., 2011 [23]; Levy et al., 2005 [24]; Robbins et al., 2004 [25]; Saxena et al., 2003 [2].
Hormone analogs (8)	Ackermann et al., 2012 [26]; Furthner et al., 2020 [27]; Gimenez et al., 2009 [28]; Goericke-Lopez Merlo et al., 2016 [29], Pesch et al., 2013 [30]; Graham et al., 2004 [31]; Rubion and Driancourt, 2009 [32]; Toydemir et al., 2012 [33].
Other contraception methods (1)	Vansandt et al., 2023 [1]
**Female Dogs**	
Vaccines (8)	Bender et al., 2009 [34]; Esmailnejad et al., 2020 [35]; Gupta et al., 2022 [36]; Liu et al., 2015 [37]; Mahi-Brown et al., 1985 [38]; Mahi-Brown et al., 1982 [39]; Saxena et al., 2002 [40]; Vargas-Pino et al., 2013 [41].
Hormone analogs (19)	Beijerink et al., 2008 [42]; Brändli et al., 2021 [43]; Corrada et al., 2006 [44]; Gontier et al., 2022 [45]; Jurczak et al., 2020 [46]; Kaya et al., 2017 [47]; Kaya et al., 2015 [48]; Lacoste et al., 1989 [49]; Marino et al., 2014 [50]; Peña-Corona et al., 2019 [51]; Peña-Corona et al., 2022 [52]; Romagnoli et al., 2009 [53]; Rubion et al., 2006 [54]; Sahara et al., 1993 [55]; Sawada et al., 1992 [56]; Schäfer-Somi et al., 2018 [57]; Sung et al., 2006 [58]; Valiente et al., 2009 [59]; Valiente et al., 2009 [60].
Compounds that regulate the CL function (6)	Concannon et al., 1987 [61]; De Gier et al., 2011 [62]; Janowski et al., 2014 [63]; Kowalewski et al., 2015 [64]; Onclin and Verstegen, 1997 [65]; Pereira et al., 2019 [66].
Kisspeptin (4)	Albers-Wolthers et al., 2016 [67]; Albers-Wolthers et al., 2017 [68]; Albers-Wolthers et al., 2014 [69]; Terse et al., 2021 [70].
Other contraception methods (2)	Mogheiseh et al., 2017 [71]; Rajabi et al., 2023 [72]

**Table 2:** Summary of contraception vaccines tested in female cats and dogs.

Age, Breed (n = Number of Animals)	Previous Treatment	Compound	Doses Administration	Administration Protocol	Main Outcome	Ref.
**Vaccines Tested in Female Cats**						
8-month-old anestrous domestic cats (n = 9)	Quarantined for 5 to 8 days, a light:dark schedule of 8:16 h; any male cats nearby	Bovine (LH-R)	0.5 mg of LH-R per implant; boost via i.m. with 0.1 mg LH-R; control: NaCl 0.9% solution	The implants were inserted on day 0; boost: on days 98, 139, 160, and 193; on day 345, LH was administered to confirm infertility	The estrus cycle of cats had normal behavior; still, inhibition of LH-R was 50 to 70% due to anti-LH-R antibodies, which was associated with suppression of serum P4, possibly indicating poor CL function	[2]
Adult in estrus shorthair cats (n = 20)	Housed at 21–23 °C for 30 days in a light:dark cycle of 12:12 h	GonaCon^TM^	200 μg of GnRH in an inactivated *Mycobacterium avium*; control: all components except GnRH-KLH	A single i.m. dose; 120 days later, a male cat was introduced	All control cats got pregnant; vaccinated cats were infertile for 1–5 years with antibody titers > 16,000; long-term infertile cats had an antibodies titer of 256,000	[23]
Domestic, short-, or long-hair cats (n = 30)	Housed at 20–27 °C and natural photoperiod	GonaCon^TM^	0.5 mL of GonaCon^TM^; control: 0.5 mL of sterile saline solution	A single dose; four months later, fertile male cats were introduced	70% of vaccinated cats got pregnant within the first year of the treatment; the estrus cycle was not altered	[20]
15- to 20-week-old cats (n = 15, 3 per group)	Housed at 21 to 23 °C with a light:dark cycle of 14:10 h	SpayVac^TM^ vaccine based on pZP antigens	50 μg of SIZP from cow or 100 μg of SIZP from cat, ferret, dog, and mink, encapsulated in liposomes, emulsified in Freund’s complete adjuvant	A single dose; 20 weeks later; cats immunized with feline ZP were boosted after 32 weeks and 5 weeks later were bred again	All cats produced antibodies; immunogenicity: mink > ferret > dog > cat; all cats got pregnant after breeding	[24]
8- to 12-week-old cats, specific pathogen-free kittens (n = 30)	Housed at a temperature around 21–23 °C with a light:dark cycle of 14:10 h	SpayVac^TM^	200 μg of SIPZ in liposomes and complete Freund’s adjuvant or alum; control: all components except SIPZ	I.m. dose under anesthesia, with a breeding trial 3 months later	Antibodies of IgG anti-porcine ZP were produced within 4 weeks; neither formulation prevented estrus cycling at maturity or reduced fertility	[21]
Domestic 2–3-year-old cats (n = 5–8 per group)	Housed next to a male, separated by wire mesh partitions, at 23 °C, light:dark cycle of 12:12 h	ZP polypeptide (55 kDa) and feline ZP A, B, and C subunits expressed by plasmid vectors	75 μg of pZP emulsified in Freund’s incomplete adjuvant for first immunization and complete for boosts; DNA vaccine expressing autologous feline ZP A, B, and C subunits; control: protoprep gel without ZP	Three boosts s.c. at weeks 0, 4, and 8; DNA feline ZP i.m. A, B, and C subunits and control group; three boosts at 1-month intervals	All cats produced IgG antibodies without feline ZP reactivity at week 10 and got pregnant; cats vaccinated with feline ZP A, B, and C developed low antibody titers until weeks 4 and 11; no significant difference in contraception for either number of follicles between the control and DNA-vaccinated groups	[19]
Domestic cats (n = 3 in group 1 and 5 in group 2)	Housed in groups of 2 or 3 under natural light conditions for 10 to 14 days before immunization	Solubilized porcine ZP	300 or 400 μg of pZP (in complete Freund’s adjuvant for the first dose and incomplete Freund’s adjuvant for boosts) in PBS and 2.0 mg of FSH and hCG; control: adjuvant and PBS	(1) Half of 300 μg injected s.c. divided in 4 injections at a 10-day interval; the missing quantity was administered on day 150; (2) half of 400 μg was administered i.m., divided in 4 injections at a 2-week interval; the missing quantity was injected 92 days after the first injection	Anti-ZP antibodies inhibit sperm binding to oocytes in vitro; a cat with the lowest antibody titer became pregnant even though it inhibited sperm binding in vitro	[22]
Domestic cats of 8–9 weeks (n = 15)	Acclimated 2 weeks prior to the study in an environmentally enriched room, at 22 ± 2 °C, with a light:dark cycle of 12:12 h	IPS-21, a recombinant fusion protein of eight tandem repeats of GnRH	10 or 100 μg of IPS-21, combined with phosphate-buffered saline, adjuvant EMULSIGEN, and dimethyl dioctadecyl ammonium bromide as an immunostimulant; control: without IPS-21 antigen	Immunizations s.c. on days 0, 48, and 243	All immunized cats developed anti-GnRH antibodies 7 days after the second immunization; the third immunization stimulated an increase in antibody titers; any female immunized showed neither signs of estrous nor became pregnant; they were anestrous due to the low P_4_ serum levels	[25]
**Vaccines Tested in Female Dogs**						
Mixed-breed and -aged bitches, in late metaestrus or anestrus (n = 14)	Some had previous parity	Crude (cCP) or pure (pCP) porcine ZP	0.4 mg of cZP or pure pZP in 0.5 mL of 0.9% NaCl solution or Tris Buffer and different adjuvants as Freund’s, Al(OH)_3_, or CP-20	s.c. (with Al (OH)_3_ as adjuvant) or i.m. (immunized with CP-20), around four times in a monthly interval	Immunized bitches turned out infertile regardless of the adjuvant	[38]
Mongrel bitches of 1–3 years, mid-metestrus to anestrus until proestrus (n = 7)	Housed in pairs indoor–outdoor; four had previously whelped	Porcine or canine ZP	2000 isolated and solubilized either porcine or canine ZP in 0.5 mL of PBS and 0.5 mL of Freund’s complete adjuvant; the boost contained 1000 ZP and Freund’s incomplete adjuvant instead	First immunization i.m. with subsequent monthly s.c. boosters until the elevation of titer antibodies had no more elevation; control bitches were treated identically, omitting ZP	ZP-treated groups developed antibodies; the pZP group had higher titles of antibodies and abnormal estrus cycles	[39]
Male and female, Chinese, rural, 16-month-old dogs (n = 6, 3 per group)	Housed individually in cages 4 weeks prior	Recombinant GnRH-I protein	1 mL of an emulsion of 1.5 mg of GnRH-I with a maltose-binding protein (MBP) emulsified in 3 mL of PBS and 3 mL of Al(OH)_3_ adjuvant; control: MBP and adjuvant	I.m at 16 months old and after 6 weeks	Immunized dogs developed antibodies; serum P4 levels decreased	[37]
Female dogs of mixed breeds over 5 months of age (n = 18, 6 per group)	From dog round-ups; dogs were separated into kennels and under oversight by a veterinarian	GonaCon^TM^	0.5 mL GonaCon^TM^ which contains 500 μg of GnRH conjugated to KLH and 85 μg of Mycobacterium avium; control: 1 mL of rabies commercial vaccine DEFENSOR-3 with inactivated virus	Group 1: GonaCon^TM^ injected i.m.; group 2: DEFENSOR-3 i.m.; group 3: GonaCon^TM^ and DEFENSOR-3 vaccines injected i.m	Antibody levels increased in three groups; results in group 3 show that rabies and immunocontraception vaccines could be administered together	[34]
Female beagle in proestrus, 1–3-year-old dogs (n = 12, 4 per group)	-	Porcine ZP3, ZP4	1 mL of a physical mixture 1:1 (500 μg of each protein): porcine ZP 3 with promiscuous T cell epitope of tetanus toxoid (TT-KK-pZP3); porcine ZP 4 with promiscuous T cell epitope of RNAase (bRNase-KK-pZP4); recombinant fusion protein encompassing dog ZP3 fragment and two copies of GnRH with appropriate promiscuous T cell epitopes (dZP3-GnRH2); control: only adjuvant	I.m. thrice every 4 weeks for each protein; a fourth boost on day 383	The groups immunized with recombinant proteins produced high antibodies titer against GnRH and ZP, especially those immunized with dZP3-GnRH2; the antibodies produced reacted specifically with dog ZP; the group immunized with dZP3-GnRH2 had the lowest number of pregnancy outcomes	[36]
Mature, mixed-breed bitches in anestrus (n = 15, 5 per group)	Antiparasitic treatment with praziquantel and mebendazole 2 weeks prior	Sperm	1 mL of high dose of sperm vaccine (200 × 10^6^ cells/mL); 1 mL of low dose of sperm vaccine (100 × 10^6^ cells/mL); boost doses just switched adjuvant for Freund’s incomplete	First immunization s.c. in four sites across the shoulder area; boosters were adminstered at weeks 1, 2, 4, and 6	Bitches immunized developed specific anti-sperm antibodies; those injected with a high dose achieved a higher titer (after the third immunization) than those injected with a low dose; there was not any dominant follicle nor active in CL	[35]
9-month-old female dogs (beagles, in the fifth day of vaginal bleeding (probably proestrus)), OM 8 to 12 kg (n = 10, 7 in the trial group and 3 in the control group)	Maintained for 5 to 8 days, alone or with compatible running partners on 12:12 h light:dark cycles	LH-R	First immunization: 0.5 mg/per implant of 100 μL of LH-R encapsulated in a silastic subdermal implant emulsified with ‘Gerbu’ as the adjuvant (1.44 mg/per implant); control: saline solution emulsified in the adjuvant; boost injection: 0.1 mg of LH-R in the adjuvant	The first immunization (week 0) was administered in a subdermal implant; oat week 14, the booster was administered by i.m. and 3 more boosters were administered at weeks 19, 22, and 27 post-implantation	Antibodies were detected in serum 3 weeks after the first immunization; serum P4 levels decreased because of a lack of ovulation and CL function; dogs remained in the diestrus and anestrus phase; serum estradiol levels remained in both the immunized and the control dogs; there were not any signs of “standing heat” nor vaginal bleeding and failed ovulation when induced by LH-RH	[40]
Twenty female dogs of mixed breeds, medium or large	Held for a 60-day observation and adaptation period	GonaCon™	Each 0.5 mL dose contained 500 μg of the GnRH conjugate and 21 μg of inactivated Mycobacterium avium in the adjuvant	GonaCon™ vaccines were administered once i.m. in the upper-left hind leg	Significant increases in anti-GnRH antibodies; P4 was suppressed in comparison to controls	[41]

Abbreviations: (OH)_3_ (aluminum hydroxide), bw (body weight), CL (corpus luteum), CP-20 (CP-20 adjuvant), cZP (crude porcine zona pellucida), eCG (equine chorionic gonadotropin), E2 (estradiol), FSH (follicle-stimulating hormone), GnRH (gonadotropin-releasing hormone), IgG (immunoglobulin G), i.m. (intramuscular), LH (luteinizing hormone), LH-R (luteinizing hormone receptor), LH-RH (luteinizing hormone-releasing hormone), mL (milliliters), n (number of animals), NaCl (sodium chloride), PL (placebo), pZP (pure porcine zona pellucida), s.c. (subcutaneous), SIZP (soluble isolated zona pellucida), STAR (steroidogenic acute regulatory protein), TT-KK-pZP3 (promiscuous T cell epitope of tetanus toxoid conjugated with pZP3), and ZP (zona pellucida).

**Table 3:** Summary of contraceptive hormone analogs tested in female cats and dogs.

Age, Breed (*n* = Number of Animals)	Previous Treatment	Compound	Doses Administration	Administration Protocol	Main Outcome	Ref.
**Hormone Analogs Tested in Female Cats**						
Queens in estrus between 2 to 5 years (n = 20, 10 per group)	Acclimated 2 months prior with natural daylight	GnRH analog	Suprelorin 4.7 mg implant (Virbac^®^)	Administration by s.c. injection; group A: treated 3.2 ± 0.8 days after the beginning of estrus; group B: treated 7 days after the end of estrus	Group A: the estrus stopped at 4.1 ± 2.5 days after treatment; estrus induction was observed 6, 138, and 155 days after treatment; group B: increase of E2 and P4 levels greater than group A one day after treatment; eight cats from this group got pregnant after mating trials	[30]
Purebred cats in proestrus; Melovine in interestrus (n = 140/83 females)	-	GnRH analog	Suprelorin 4.7 mg implant (Virbac^®^), 18 mg of melatonin implant (Melovine^®^)	41 and 42 queens were implanted s.c. with deslorelin and melatonin, respectively	26/41 female cats implanted had estrus inhibited for 8–38 months; 12/26 queens produced a litter; 33/42 queens implanted with melatonin had estrus inhibited for 21–277 days after implantation; 12/33 queens had a subsequent litter	[27]
Mature (2–3 years old) mixed-breed domestic queens in interestrus or diestrus (n = 10)	Kept in an experimental cattery, with light exposure of 12 h/day	GnRH analog	Implant containing 4.7 mg of deslorelin acetate	Implanted s.c. on day 0; on day 90, they were removed; on day 100, estrus and ovulation were induced with eCG (i.m.), followed by hCG (i.m.) 84 h later	After deslorelin application, 40% of queens ovulated; 4 queens had signs of estrus, but just 1 ovulated; 3 queens showed ovulation did not have signs consistent with estrus; mean plasma P4 levels decreased gradually after placement, and rapidly increased after removal	[26]
Female, short-haired, mixed-breed, 1–5-year-old, domestic cats (n = 28)	The queens had previously experienced 6–10 estrus cycles; 12 of them had litters beforehand and the fertility of the rest was unknown; kept at 15–17 °C and 12:12 h of light:dark cycles	GnRH analog and MA	Group 1 (G1): 9.5 mg deslorelin implant; group 2 (G2): 9.5 mg of deslorelin implant and 5 mg of MA tablets; control group: placebo implants	Implants in G1 were administered s.c.; in G2, implants were administered together with MA 14 days and 12 h before and 14 days after implants; they remained for 18.5 months and were ovariectomized	Fecal E2 levels were significantly lower in treatment groups (especially the G2 after one day) than the control group; most of the queens treated did not have estrus behavior for 18.5 months; queens that showed estrus behaviors after implantation did not become pregnant; the ovaries from G1 and G2 had no presence of CL nor fewer follicles and uterine thickness was lower	[33]
1- to 4-year-old queens and tomcats (n = 12, 6 per group)	14:10 h of light:dark cycles	GnRH agonist	Implant containing 20 mg of Gonazon	s.c. on the neck, and it remained for 3 years	Gonazon inhibited ovulation in all treated queens; Gonazon concentrations peaked after a week of the implant insertion, remained for a month, and decreased slowly	[32]
Domestic short-hair 8- to 18-month-old queens (n = 12)	Hosted in pairs under artificial fluorescent lighting (12:12 h light:dark cycle at 7:00 p.m.)	Melatonin	30 mg of melatonin; ovarian stimulation (OS): 100 IU eCG i.m. to stimulate follicular development, followed by 75 IU hCG i.m. 80 h later to induce final oocyte maturation and ovulation	Experiment 1 (n = 4): oral capsule administration; experiment 2 (n = 6): oral administration for 35 days, 3 h before lights off; experiment 3 (n = 5 per group): OS by eCG/hCG only, or 2 days after pre-treatment with oral melatonin with eCG administration; experiment 4 (n = 6 per group): examination after AI, in queens with OS	3/6 melatonin-treated cats had elevated fecal E2 levels during treatment; their estrus cycle returned after cessation; treatment with melatonin and eCG reduced ancillary follicle development and had no significant effect on the quantity or quality of AI-produced embryos; fecal E2 levels were lower in queens pre-treated with melatonin than the only-eCG-treated with no significant difference; melatonin inhibited ovarian activity without adverse impact on embryo quality after artificial insemination	[31]
Mature (12–14 months) female cats in interestrus and estrus (n = 9 in the trial group; 5 in the placebo group)	Kept in cages for 45 days under artificial illumination in 14:10 h of light:dark cycles	Melatonin	18 mg melatonin implant, placebo without melatonin	Placed s.c. in the subsequent estrus, all received a second implant; a male was introduced in the following estrus after the second dose	Melatonin implants suppress estrus for 2–4 months; within 9 to 11 d after melatonin implant insertion during estrus, 78% of queens had estrous behavior; 75% of melatonin-implanted cats became pregnant	[28]
Postnatal female kittens (n = 10)	Indoor catteries with 14 h of light per day and weaned at the age of 40 days	Medroxyprogesterone acetate	10 mg/animal of medroxyprogesterone acetate (MPA) (n = 6); placebo: 0.2 mL of corn oil (n = 4)	Within the first 24 h of birth, kittens were randomly assigned to treatment, which was administered subcutaneously	Ovulation occurred in 4/6 MPA-treated kittens and 3/4 from placebo group after estrus and became pregnant; all pubertal kittens showed normal sexual behavior and when exposed to males during estrus accepted repeated matings	[29]
**Hormone Analogs Tested in** **Female Dogs**						
Cross-breed, medium-sized, prepubertal bitches (4–5 months old) (n = 13, 5 in group 1, 4 in group 2 and placebo group)	Housed in indoor–outdoor runs, 12 h daylight exposure	GnRH analog	Implants with Suprelorin (deslorelin acetate): group 1: 9.4 mg, group 2: 4.7 mg, group 3: placebo (sodium chloride 0.9%)	s.c. insertions in the interscapular region	Half of the bitches developed estrus after 83 and 102 weeks Serum levels of estradiol 17β increased at weeks 37 to 49 in 3 bitches in each group; there was not any significant change 40 weeks of treatment	[48]
Healthy, caucasian shepherd and kangal cross-breed, prepubertal bitches aged 4 months and 9 kg in body weight (n = 13)	-	GnRH analog	9.4 mg of deslorelin acetate implant (n = 5), 4.7 mg of deslorelin acetate implant (n = 4), 0.9% sodium chloride as placebo (n = 4)	s.c. single-use administration; the animals that showed estrus signs were ovariohysterectomized during the mid-luteal phase, and mature corpora lutea were collected	The expression of prostaglandin E2 receptors was significantly higher in the CL of deslorelin-treated bitches; the vascular endothelial growth factor receptor VEGFR1 increased significantly in CL	[47]
Bitches younger than 4.5 years (n = 32)	Client-owned	GnRH analog	9.4 mg of deslorelin acetate implant (n = 5), 4.7 mg of deslorelin acetate implant (n = 4), 0.9% sodium chloride as placebo (n = 4)	1st treatment: 4.7 mg, n = 20 and 9.4 mg, n = 12; subsequent treatments: 9.4 mg, n = 21 and 4.7 mg, n = 11; bitches had the implant for 0.5–11.3 years	Long-term side effects in 3 dogs, after more than five years of treatment, were persistent heat, ovarian tumors, and cystic endometrial hyperplasia; flare-up was an immediate side effect	[43]
Cross-breed prepubertal bitches 4.2 ± 0.6 months of age (n = 11)	Housed in indoor–outdoor runs with average of 12 h daylight exposure	GnRH analog	4.7 mg Suprelorin implant (n = 3), 9.4 mg Suprelorin implant (n = 4), sodium chloride 0.9% (placebo) (n = 4)	s.c., animals were ovariohysterectomized before puberty or metestrus	All from the placebo group and two from Suprelorin implant came into estrus; no significant difference in serum P4 and E2 levels between the placebo and Suprelorin treatments	[57]
Healthy in anestrus, 20–35 kg, 7-year-old pure-breed bitches (n = 42)	-	GnRH analog and synthetic progestin	10 mg of DA biocompatible implant, 2 mg/kg of MA slotted of 20 mg tablets, PL implant formulated as the inert matrix of DA without drug	s.c. DA and PL and MA oral administration; PL (n = 12) once; MA (n = 4) for 8 days; DA (n = 8) once; MA and DA-1 (n = 8): MA beginning one day before DA; MA and DA (n = 10): MA beginning 4 days before DA	Interestrous interval post-treatment of DA-treated bitches was longer than the PL-treated bitches; simultaneous administration of MA with DA did not seem to significantly prolong the effect of DA	[44]
Mixed-breed bitches in anestrus aged 5, 4, and 4 years with normal reproductive history and progesterone levels <1 ng/mL (n = 3)	-	GnRH analog and 3β-hydroxysteroid dehydrogenase inhibitor (trilostane (Vetoryl^®^))	4.7 mg of Suprelorin^®^, 10 mg/kg body weight of trilostane (Vetoryl^®^)	Vetoryl^®^ 6 h before and in deslorelin implant application, and in at 6 and 9 h after; the Suprelorin^®^ implant was introduced simultaneously with the second administration of Vetoryl^®^ s.c.	The serum levels of LH, P4, cortisol, and E2 decreased through the first 4 administrations of Vetoryl^®^; P4 serum levels decreased, and 3 bitches developed signs of estrus or diestrus around day 16 after implantation of Suprelorin	[46]
Around 5 months of age, beagle bitches (n = 20, 10 per group)	-	GnRH agonist	18.5 mg of azagly-nafarelin (Gonazon^®^) or placebo	s.c. in the umbilical region and were kept for 1 year	None of the bitches treated with Gonazon^®^ displayed estrus or ovulation during the treatment, just after the implant was removed	[54]
12–18 weeks old, mixed breeds, prepubertal bitches (n = 83)	-	GnRH analog	4.7 mg of Suprelorin implant (n = 62), 1 mL of 0.9% sodium chloride (n = 21)	Implants were administered s.c.	The Suprelorin group had a delayed estrus compared to the PL (377 days vs. 217 days); after 24 months post-implantation, all bitches from both groups showed functional reproductive abilities	[45]
Sicilian hound female dogs (n = 24)	-	GnRH analog	4.7 mg of DA implant; control: non-implanted animals	Treatment group: at 4.5, 9, and 13.5 months; control: ovariohysterectomized at 4.5 or 18 months	The estrus was suppressed in the treated group after the first implantation; E2 and P4 levels remained at baseline; there were no follicular structures	[50]
Adult healthy (n = 7) or with mammary neoplasia bitches (n = 3) in anestrus or diestrus	Absent of irregular estrus cycles and of prolonged or abnormal vulvar discharge	GnRH analog	4.7 mg or 9.4 mg of DA implant	s.c., the treatment was repeated every 5 months depending on the dogs’ requirements	Bitches implanted during anestrus came in heat 4–15 days after treatment; the 9.4 mg implant had estrus inhibition for 11–14 months; the 4.7 mg implant appeared to perform for only 5 months; suppression of reproductive cyclicity was achieved in 60% of bitches for 1–4 years	[53]
Healthy adult 1–3-year-old greyhound bitches, in anestrus (n = 15)	-	GnRH analog	Deslorelin implant and 14 doses of 2 mg/kg MA (n = 5); placebo group untreated (n = 10)	Deslorelin implant on day 0; the MA group was treated orally daily from day −7 to 6.	In treated group, estrus signs were observed and plasma LH and E2 were lower	[58]
Prepubertal beagle dogs (n = 25, where half of them were female)	Controlled conditions and light exposure from 6:00 to 18 h	GnRH ethylamide	100 μg of [D-Trp6, des-Gly-NH210] GnRH ethylamide dissolved in 0.9% NaCl-1% gelatin (n = 9 females); vehicle only for the control group (n = 8 females)	Subcutaneous injection daily for 23 months	Treatment inhibits sexual maturation and after cessation of it, normal pituitary–gonadal functions resume normally	[49]
Beagle bitches in anestrus aged 3–9 years (n = 5)	-	Synthetic progestin	10 mg/kg bw MPA	s.c. administration at intervals of 4 weeks for a total of 13 times	No signs of estrus during the 12 months of treatment	[42]
Sexually mature female dogs in anestrus (n = 15)	-	Phytoestrogens	600 μg of coumestrol per kg of bw diluted in 20 μL of DMSO (n = 5); commercial food biscuit was given as a placebo with 20 μL of DMSO (n = 5)	Single or administration in a dog food biscuit	Coumestrol increased serum E2 levels on days 21 and 28; the number of vaginal cells remained in anestrus bitches; DMSO: increased serum P4, vaginal anucleated superficial cells, and diestrus length	[51]
Sexually mature female dogs in estrus (n = 15)	-	Phytoestrogens	600 μg of coumestrol per kg of bw diluted in 20 μL of DMSO (n = 6); commercial food biscuit was given as a placebo with 20 μL of DMSO (n = 5)	Single or administration in a dog food biscuit	In animals that ovulated, COU did not affect hormonal profiles; in contrast, animals that did not ovulate showed lower circulating P4 concentrations	[52]
Domestic purebred bitches in anestrus (n = 19)	-	GnRH analog + GnRH antagonists	DA 10 mg implant (n = 6), DA 10 mg + and 330 mg/kg of acyline as a lyophilized powder suspended in water (2 mg/mL)	DA administration s.c.; DA + acyline was implanted s.c. and 48 h later, acyline was administered s.c.	All bitches developed estrus for the first month; the interestrus intervals were not significantly different; 6 bitches had an estrus response and ovulated with DA administration	[59]
Postpubertal bitches in proestrus (n = 20)	-	GnRH antagonists	Respective volume of acyline 2.2 mg/mL suspension, 110 μg/kg of acyline (n = 6), 330 μg/kg of acyline (n = 8); placebo (n = 6)	Injection s.c.	Estrus cycles and ovulation of treated groups were interrupted efficiently and reversibly for about 3 days; there was not a significant difference between both doses and return to estrus periods	[60]
Mature (1–4 years old) and immature (6–7 months old) bitches, 4 months after the proestrus period (n = 24 and 3, respectively)	-	Synthetic progesterone	4–12.5 mg/kg of chlormadinone acetate; 2–6.25 mg/kg and 2 mg/kg of chlormadinone acetate	Twice a year: chlormadinone acetate to mature bitches daily for 7 days.; oral administration of chlormadinone acetate to mature and immature bitches	Bitches with the twice-a-year treatment had no long-term effect on estrus prevention activity after the end of the treatment, compared to the once-a-week-treated, which had an effect for 1 year or longer	[56]
Mongrel bitches aged from 8–48 months (n = 25, divided randomly into 5 groups); anestrus approximately 5 months after the last estrus (n = 21), and the beginning of proestrus (n = 4)	-	Synthetic progesterone	2.5, 5, 10, 25, or 30 mg/kg of CA and silastic silicone rubber with a coagulant implant	s.c. administration; experiment 1: 19 bitches with 2.5–10 mg/kg doses, the implants were left for 24 months; experiment 2: the implants were removed to 6 bitches, 3 of them were given doses of 10, 20, and 30 mg/kg, and implants were removed after 12, 12, and 17 months; the 3 bitches left were given a 10 mg/kg dose implant for 29, 28, and 48 months	Estrus was observed after 3–13 months of administration in bitches with 2.5 mg/kg dose, 13–15 months in 3/5 bitches with 5 mg/kg dose, and more than 24 months in bitches with doses of 10 mg/kg and more; bitches in experiment 2, given the 10, 20, and 30 mg/kg doses, came into estrus 4, 8, and 10 months and the second estrus after 10, 14, and 18 months after removing the implant, respectively	[55]

Abbreviations: 3β-HSD (3β-hydroxysteroid-dehydrogenase), ALHS (anti-LH serum), bw (body weight), CA (chlormadinone acetate), CL (corpus luteum), cZP (crude porcine zona pellucida), DMSO (dimethyl sulfoxide), eCG (equine chorionic gonadotropin), FSH (follicle-stimulating hormone), GnRH (gonadotropin-releasing hormone), hCG (human chorionic gonadotropin), i.m. (intramuscular), i.v. (intravenous), LH (luteinizing hormone), LH-R (luteinizing hormone receptor), LH-RH (luteinizing hormone-releasing hormone), MA (megestrol acetate), mL (milliliters), NaCl (sodium chloride), n (number of animals), PDP (pituitary-dependent part), PGE2 (prostaglandin E2), PIP (pituitary-independent part of the luteal phase), PL (placebo), pZP (pure porcine zona pellucida), P4 (progesterone), s.c. (subcutaneous), STAR (steroidogenic acute regulatory protein), and ZP (zona pellucida).

**Table 4:** Summary of compounds that regulate the CL function as contraceptive tested in bitches.

Age, Breed (*n* = Number of Animals)	Previous Treatment	Compound	Doses Administration	Administration Protocol	Main Outcome	Ref.
**Compounds That Regulate the CL Function Tested in** **Female Dogs**						
Beagle bitches (n = 16, 6 per group), probably estrus	-	3β-HSD inhibitor	4.5 mg/kg of trilostane	Administered orally twice a day for seven days, beginning on day 11 (PIP, n = 6) or 31 days after ovulation (PDP, n = 6)	Trilostane decreased P4 plasma concentration, so plasma prolactin did not increase	[62]
2–4-year-old beagle bitches in luteal phase (n = 33) (probably diestrus)	Maintained in individual cages under a 12 h light:12 h dark schedule	ALHS dopamine agonist (bromocriptine)	10 mL of equine ALHS (n = 5, where 2 were pregnant and 3 non-pregnant), 0.1 mg/kg bw of bromocriptine (n = 11); normal horse serum in ALHS control group (n = 6, where 3 were pregnant and 3 non-pregnant) and 20% ethanol in saline for bromocriptine control group (n = 11)	Single i.m. injection of ALHS and its control, at day 42 of pregnancy or ovarian cycle; bromocriptine and control were injected i.m. daily for 6 days starting on day 8, 22, or 42 of pregnancy and day 42 of ovarian cycle of non-pregnant bitches	ALHS reduced P4 levels in all treated bitches by 24 h but returned levels on day 4 after injection; bromocriptine caused abrupt declines in P4 in all treated bitches at day 8 after injection	[61]
Mature, pregnant, and non-pregnant beagle bitches (n = 30)	Indoor–outdoor runs in groups (2–5) with natural light exposure	Cabergoline	Prolactin (375 μg/animal/injection) and cabergoline or LH (750 μg/animal/injection) and cabergoline; placebo: propylene glycol	Subcutaneous administration of cabergoline 30 days after LH surge (10 pregnant, 10 non-pregnant)	Plasma prolactin levels were suppressed, and so progesterone for 5 days and non-effect of LH was observed after cabergoline injection	[65]
Mixed-breed and -age (2–7 years) bitches in diestrus (n = 30)	-	Firocoxib	Treatment group: 10 mg firocoxib/kg bw per day; control Group: placebo	Administration for 5, 10, 20, or 30 days from day 0 (the day of ovulation)	Decreased expression of 3β-HSD mRNA and protein, area of the luteal cell nuclei, P4 concentrations	[63]
Mixed-breed 2–7-year-old bitches in diestrus (n = 30)	-	Firocoxib	10 mg/kg bw daily dose of firocoxib; untreated dogs served as controls	Ovariohysterectomies were performed on days 5, 10, 20, or 30 depending on the treatment group	Firocoxib decreased the mRNA expression of STAR protein, PGE2 synthase, and prolactin receptor	[64]
Middle-sized, mixed-breed, 2–7 years of age, diestrus (n = 37).	-	Firocoxib	10 mg/kg body weight per day of firocoxib; control: PL	Oral administration for 5, 10, 20, and 30 days (first day of ovulation = day 0); bitches were ovariohysterectomized on the last day for collection of CL	Firocoxib inhibited cyclooxygenase 2/prostaglandin synthase 2, which caused PGE2 decrease, which suggests their matter in regulating CL	[66]

Abbreviations: 3β-HSD (3β-hydroxysteroid-dehydrogenase), ALHS (anti-LH serum), bw (body weight), CL (corpus luteum), cZP (crude porcine zona pellucida), FSH (follicle-stimulating hormone), GnRH (gonadotropin-releasing hormone), i.m. (intramuscular), i.v. (intravenous), LH (luteinizing hormone), mL (milliliters), NaCl (sodium chloride), n (number of animals), PDP (pituitary-dependent part), PGE2 (prostaglandin E2), PIP (pituitary-independent part of the luteal phase), PL (placebo), pZP (pure porcine zona pellucida), s.c. (subcutaneous), STAR (steroidogenic acute regulatory protein).

**Table 5:** Summary of studies administered kisspeptin as a contraceptive method in bitches.

Age, Breed (*n* = Number of Animals)	Previous Treatment	Doses Administration	Administration Protocol	Main Outcome	Ref.
**Kisspeptin (KP) Administration** **Tested in Female Dogs**					
Adult 86 and 41 months of age, 14–15 kg bw beagle bitches in the follicular phase (n = 12)	Were housed in pairs in indoor–outdoor runs	Experiment 1: 0.5 μg/kg bw of canine kisspeptin-10 (KP-10); experiment 2: 0.5 μg/kg body weight of canine KP-10 and an infusion with 50 μg/kg body weight per hour of p271	I.v. administration in the cephalic vein; in group 2, a continuous i.v. infusion with p271 was administered for 3 h in the cephalic vein, and canine KP-10, 2 h after the start of the p271 infusion	Canine KP-10 increased plasma LH concentration and peaked 10 min after administration; p271 did not alter the LH concentration nor lower the LH response to KP-10	[67]
Healthy, median age of 36–70-month-old beagle bitches, in anestrus (n = 12, 6 per group)	Housed in pairs in indoor–outdoor runs	High-dosage group: 1–30 μg/kg canine KP-10; low-dosage group: 0–1 μg/kg canine KP-10	30 μg/kg and next doses were decreasing to 10, 5, 1, and 0 μg/kg or in the reverse order by the cephalic vein at weekly intervals during anestrus	LH secretion peaked 10 min after administration; high-dosage group increased the plasma levels of LH, E2, and FSH; low-dosage group: increase in LH plasma level	[69]
Beagles, 8–12-month-old dogs in anestrus (n = 20)	-	2.6 mL/kg of 30, 100, and 1000 μg/kg of KP-10 or sterile saline solution (control) once a day	Daily i.v. for 14 consecutive days with a 14-day recovery period; necropsied 15 (n = 3 per group) and 29 days (n = 2) after 14-day dosing	Bitches remained in the anestrus phase	[70]
Beagle female dogs in anestrus (n = 13)	-	Control (n = 6): 0.5 μg/kg canine KP-10; antagonist experiments (n = 8): canine KP-10 and 50 μg/kg/h antagonists; 4 bitches received p271, p354, and p356; 2 bitches received p354 and p356; and 2 bitches received only p271	Continuous administration of KP-10 antagonists for 3 h via a catheter in the cephalic vein; two hours later, canine KP10 was administered i.v.; the time between testing of different antagonists was 5–15 days	None of these antagonists lowered the basal plasma LH concentration and none of the peptides lowered the KP10-induced LH response	[68]

Abbreviations: CL (corpus luteum), FSH (follicle-stimulating hormone), GnRH (gonadotropin-releasing hormone), i.m. (intramuscular), i.v. (intravenous), KP-10 (kisspeptin-10), LH (luteinizing hormone), mL (milliliters), NaCl (sodium chloride), s.c. (subcutaneous), ZP (zona pellucida).

**Table 6:** Summary of other contraceptive methods tested in female cats and dogs.

Age, Breed (*n* = Number of Animals)	Previous Treatment	Doses Administration	Administration Protocol	Main Outcome	Ref.
** Other Contraceptive Methods Tested in Female Cats **					
Domestic short-haired cats (Felis silvestris catus), nulliparous, sexually intact (main study n = 9, pilot study n = 3)	Physical examinations and blood tests	A single intramuscular injection of the AAV9 viral vector carrying the fcMISv2 transgene, which encodes feline anti-Müllerian hormone; low dose: 5 × 10^12^ vg/kg; high dose: 1 × 10^13^ vg/kg; control: empty vector AAV9 (5 × 10^12^ vp/kg)	The single injection was administered into the right caudal thigh muscle; two mating trials were conducted in which females were housed with a fertile male for 4 months per trial, 8 h per day, 5 days per week	All cats in the control group became pregnant and had offspring, while no AAV9-fcMISv2-treated cats became pregnant	[1]
** Other Contraceptive Methods Tested in Female Dogs **					
Clinically healthful, mixed-breed, reproductively adult anestrous female dogs (n = 26)	Checked for pregnancy by transabdominal ultrasound	Therapeutic ultrasound 1 MHz, 1.5 W/cm^2^	Control (laparotomy) group (n = 6) and two treatment groups: 5- and 10-min treatment of ovaries with therapeutic ultrasound wave during laparotomy	Systemic inflammation, oxidative stress, decreased primordial follicles, and lower oocyte preservation score, suggesting ovarian damage and possible long-term subfertility	[72].
Healthy female adult dogs, mixed-breed (n = 5)	General clinical evaluation	Dose variable according to ovarian diameter, calculated by ultrasound scan	Direct intra-ovarian injection of neutral zinc gluconate by laparotomy with G24 needle; monitoring for 28 days	Reduction in ovarian size, increase in atretic follicles, histological changes such as fibrosis and hemorrhage; without complete destruction of ovarian tissue	[71]

## Data Availability

The original contributions presented in this study are included in the article. Further inquiries can be directed to the corresponding authors.

## Abbreviations

The following abbreviations are used in this manuscript:

AAV	adeno-associated virus
ALHS	anti-LH serum
Al(OH)^3^	aluminum hydroxide
b.w.	body weight
CL	corpus luteum
cZP	crude porcine zona pellucida
CP	complete Freund’s adjuvant
CP-20	CP-20 adjuvant
DA	deslorelin acetate
DMSO	dimethyl sulfoxide
E2	estradiol
eCG	equine chorionic gonadotropin
FSH	follicle-stimulating hormone
GnRH	gonadotropin-releasing hormone
hCG	human chorionic gonadotropin
IgG	immunoglobulin G
i.m.	intramuscular
i.v.	intravenous
KLH	keyhole limpet hemocyanin
KP-10	kisspeptin-10
LH	luteinizing hormone
LH-R	luteinizing hormone receptor
LH-RH	luteinizing hormone-releasing hormone
MA	megestrol acetate
MAL	luteal Hhrmone
mL	milliliters
NaCl	sodium chloride
P4	progesterone
PGE2	prostaglandin E2
PBS	phosphate-buffered saline
pZP	porcine zona pellucida
SIZP	soluble isolated zona pellucida
s.c.	subcutaneous
STAR	steroidogenic acute regulatory protein
TT-KK-pZP3	promiscuous T cell epitope of tetanus toxoid conjugated with pZP3
ZP	zona pellucida
